# Pericementitis Producing Abscess

**Published:** 1894-06

**Authors:** C. N. Johnson


ARTICLE VIII.

### PERICEMENTITIS PRODUCING ABSCESS.

C. N. JOHNSON, L. D. S., D. D. S.

Probably one of the most painful affections with which the dentist has to deal is that of acute pericementitis immediately preceding alveolar abscess. This is easily accounted for when we consider the anatomical relations of the tissues surrounding the root of a tooth. The pericementum is encased in a bony socket with unyielding walls on either side, and a slight degree of inflammation results in a great pressure. Whatever may cause pericementitis, there sometimes comes a stage when no kind of treatment directed into the pulp chamber and through the pulp canals is in the least effective. It is to the consideration of this phase of the affection that I wish briefly to call attention.

Where the canals have been opened up and a free vent made for the escape of gases, and this has failed to bring relief, it is an indication that the tissues of the peridental membrane have become so far implicated that the inflammatory process will go on independent of further irritation. This has always proved in my hands the most difficult stage of the affection to control, till within the past two or three years. During that time I have followed a practice which, if faithfully carried out, has seldom or never failed to give satisfactory results. The treatment consists in the application of moist heat to the gums surrounding the affected tooth.

The method of procedure is as follows: Water as hot as the tissues will tolerate is taken up in a large bulb syringe having a fine point. A jet of hot water is directed on the gums and into the cavity of the tooth, all cotton or other dressings having previously been removed from the canals. The tooth and surrounding tissues are thus submerged in water as hot as the patient can tolerate. During the refilling of the syringe the water is to be retained in the mouth and then emptied into the spittoon just previous to another application. The water should be kept on a gas stove near at hand and the heat gradually raised as the tissues will admit. It will be found that in the end water extremely hot may be used if the heat be gradually increased, and one of the essentials to success is to employ an exceedingly high degree of heat. If the tongue and other tissues of the mouth remote from the region of the tooth are too much affected by the water, a saliva ejector may be used to carry off the water after it has flown over the gum in the immediate vicinity of the inflammation. The gum in this region will often tolerate a much higher temperature than the normal tissues. In fact, an application so hot as to be extremely painful to normal tissue often proves instantly soothing to the affected parts. The process should be kept up till perfect relief has been obtained, and the length of time necessary for this varies. In some instances where the pain is most distressing prior to the application, the relief will be very sudden; in others it will require persistent treatment for perhaps thirty or even forty minutes before a substantial effect is produced. I have never yet encountered a case where persistent effort failed to finally bring a cessation of pain. The permanency of the relief is governed largely by conditions.

If the general tone of the system is bad; if there is universal congestion strongly marked by the symptoms of what is commonly called "a heavy cold;" if the circulatory and absorbent systems are badly out of condition, or if the excretory organs fail to perform their function, then the relief is necessarily temporary, and we must resort to general treatment to gain permanent relief.

A patient in this condition should be given a rapidly acting cathartic, the citrate of magnesia, in large doses, having proved in my experience the most pleasant and satisfactory. Again, if the inflammation has gone on to a point where suppuration has begun in the apical space, we cannot hope for complete relief till the abscess has pointed and discharged, but even in these cases we may be reasonably sure of preventing extensive infiltration and puffing of the superficial tissues, and also of mitigating the suffering.

As to the circumstances which may render the relief temporary, I have noted that if the patient left the office immediately after the hot water application, and went into the open air on a cold day without carefully protecting the whole side of the face from the air, the pain would ordinarily recur. In other words, exposure would bring back the trouble. But not the least among the gratifying results that have followed this line of practice in my hands is the large per cent. of cases where a permanent relief has been gained.

A criticism might be made by some as to the advisability of leaving the cavity and canals open during the treatment. The argument might be used that there was danger of additional infection through the medium of the open canals, but I never have seen a case where I could trace any evil effects to this cause. My reasons for leaving the canals open are that I wish the hot water to reach the apical space if possible, and that there may be a liklihood of the water, in flooding the cavity and canals, floating to the surface or dislodging small particles of debris that may have been packed in the canals near the apical foramen, and which the broach has failed to bring away. I never leave the cavity open when I dismiss the patient. If the tooth is not too sensitive to pressure I usually dry the canals as thoroughly as possible after the pain is relieved, and then flood them with an antiseptic. A small pledget of cotton, saturated with the antiseptic, is then placed loosely in the chamber, and the cavity sealed with Gilbert's temporary stopping.

The canals are never packed with cotton at this sitting, and if the tooth is much raised in its socket, and extremely sensitive to pressure, I do not attempt to seal with gutta percha. It should be worked on with instruments as little as possible. Mechanical irritation invariably results in increased sensitiveness.

Where the tooth is loose and sore from swelling of the pericementum, I simply dry the canals, flood them with an antiseptic, and place some cotton in the cavity, merely to keep food and debris from packing into the tooth. I should much prefer dismissing the patient in this condition and taking chances of infection through the imperfectly sealed cavity to attempting a thorough sealing at this sitting. These are mostly emergency sittings, and our principal office is to relieve the present distress. Usually in twenty-four hours the tooth will be in a condition to work on, and it may then be treated and sealed.

The simplicity of the hot water treatment would seem to argue that the case could as well be treated by the patient in his own home as by the dentist in his office, but my experience has taught me that the results are seldom satisfactory. Somehow patients do not succeed, however careful the instructions. They do not use the water hot enough, or they do not apply it properly, or do not keep up the application a sufficient length of time.

In fact, the element of time in this treatment proves one of the greatest obstacles to its practical application. As has been stated these are emergency cases, and are likely to call for attention at any hour through the day without previous appointment. In a full practice where almost every moment of every hour is systematically assigned in advance for the regular routine work of the office, it is exceedingly trying to the operator to be called away from his work to devote any considerable time to an unexpected patient. In those cases where I have fallen short of producing the most satisfactory results it has been traceable to this dilemma. And yet a dentist's first duty as a practitioner and as an individual is to relieve suffering whenever or however it appears.

In searching for a theory to account for the results obtained by the application of hot water I was lead to consult Dr. W. T. Belfield. I then learned for the first time that he had long been a strong advocate of hot applications for the relief of pain in other tissues. His theory was that heat, especially moist heat, was an anesthetic. This would account for the relief of pain temporarily, but if this were the only action of hot water the pain would soon recur. To account for the permanent results which I claimed to have undoubtedly secured in many cases, he said that hot water applied in the manner I had indicated would prove a very rapid and effectual absorbent. It is to this latter quality that I attribute the lasting results, and this argues especially for a somewhat protracted application.

Dr. Belfield also stated that in the use of hot water, a very minute stream should be used to play on the tissues. A jet, such as would come from a hypodermic needle he had found more effective than a larger stream.

In conclusion, I may add that in some cases where the pain has been excruciating, I have added to the hot water a few drops of carbolic acid, making about a five per cent solution. The anesthetic effect of carbolic acid is thus added to that of the hot water, and the relief is usually exceedingly prompt and gratifying.

DISCUSSION.

Dr. W. T. Brophy: Heat and moisture employed as a therapeutical agent for the treatment of acute inflammation was long ago recognized and highly regarded.

The merits of hot water, when properly applied to a part soon after stasis has occurred, cannot be too highly extolled. I have never employed it in the manner described by Dr. Johnson, but the advantage to be derived by applications such as he suggests commend themselves to us, because the object desired in applying heat and moisture is more speedily accomplished than by the usual methods.

Hot water applied to a part in the first stage of inflammation has the effect of dilating the vessels surrounding the inflamed territory, thereby attracting the blood from the engorged vessels at the seat of inflammation,and thus promoting the flow of blood and re-establishing the normal circulation, terminating the inflammation by resolution.

Dr. E. A. Royce: Eighteen years ago I was told to use this prescription, and for an hour I sat with a cup of hot water in my hand and my mouth full. It certainly gave me relief, but it was a tiresome operation, and I do not wonder that our patients will not persevere in its use till they gain the full benefit of the application.

A little experience in my family may serve to show the efficacy of this simple remedy in cases of inflammation. Some time ago a lady turned her foot while on a dancing floor. Severe pain followed, and she was removed to her home and the foot placed in hot water. For three hours the temperature of the water was kept as high as could be endured, and at the end of that time the patient had little or no pain and slept well. In the morning the small bones of the foot that had been misplaced were put in their proper position with but little pain and no swelling. It was a simple remedy, yet it saved the patient days of suffering.

My attempts to use hot water in my practice have not been satisfactory because of the primitive method of application, but with the methods given by Dr. Johnson I feel sure we have a wonderful remedy in our hands.

Dr. A. W. Harlan: For acute pericementitis I have used a hot pack on the neck and side of the face with three or four thicknesses of heavy toweling wrung out of hot water. At the same time I give one-tenth grain calcium sulfid, one-tenth grain every ten minutes for the first hour every fifteen for the second hour, and one every half hour for two or three hours longer. To arrest pain at night use

| | |
|---|---|
| R.—Acetanilid . . . . . . | gr. viii. |
| Syr. simple . . . . . | ʒ ij. |
| Spir. frumenti . . . . . | ℥ ij. |

M.—Sig. Take one-half the above at 5 or 6 P. M., and the remainder at 10 P. M.—*Review.*

